# Mr. Graham’s Case of Cramp in the Stomach

**Published:** 1842-05-07

**Authors:** Robert Graham

**Affiliations:** Surgeon


*Cramp in the Stomach.* By ROBERT GRAHAM, Surgeon.—About eight or nine years since I had a patient in Glasgow, a married lady, about thirty years of age. She had had a large family previous to my acquaintance with her, but had for many years been subject to violent attacks of cramp in the stomach ; on account of one of which I was first called to see her, and at which time I thought she would have died. I need not enumerate the antispasmodics which were used at that time ; having then and subsequently tried all those recommended for the complaint, without being able to say that I had succeeded in even checking the spasm for the time. It seemed eventually to wear off of itself. I had bled her, which gave relief for once ; but it was followed by such weakness, that when called to witness another attack two or three days after, I dared not repeat it.

The thought, by and by, crossed my mind, that I could produce a counter-spasm ; so I took a strong tumbler, and with a bit of lighted paper applied it as a cupping glass over the stomach, when almost immediately I had the satisfaction of hearing my patient, who could not speak a moment before, exclaim, " The pain is gone." Since that time it has invariably been a source of relief with her when attacked ; and I do not recollect of its ever failing, if a large cupping glass was applied firmly once or twice over the part.—*London Lancet.* March 12th, 1842.

